# Retention of Urine with Hæmaturia

**Published:** 1883-03

**Authors:** W. A. Hawley

**Affiliations:** Syracuse, N. Y.


### CLINICAL BUREAU.

### RETENTION OF URINE WITH HÆMATURIA.

W. A. Hawley, M. D., Syracuse, N. Y.

In the early part of June, 1882, Dr. A. J. B. came to me for a prescription, giving this history of his case: The day before was rainy and was out so as to get somewhat wet; on returning to his office he found his fire had gone out, but sat and wrote for an hour or more without rekindling it. When he got home he found himself seriously chilled, and the next morning on rising he found he could not void his water. He relieved himself with a catheter and took *Canth.*, but without relief. Considering the cold and wet as the source of the trouble, I gave him *Rhus.*$^{cc}$, and advised his going home and to bed. The next morning he sent for me. I found he had had no relief, and that the water drawn with the instrument was quite bloody. He was without thirst and with only slight fever, but complained of frequent urging to urinate and flatulent condition of stomach and bowels. I gave him *Lyc.*$^{cc}$ The next day his condition was unchanged, except that the urine was much more bloody. Considering the *thirstlessness*, bloody urine, and the retention, with constant urgency, I put him on *Puls.*$^{52m}$ (Fincke), once in three hours. The third visit I found he had voided his water, which was much less bloody. Continued the remedy. The fourth day the urine was natural and passed without difficulty. Stopped the medicine, and without anything further he resumed his business just a week from the day he first called on me.

